# Metabolomic Fingerprint of Mecp2-Deficient Mouse Cortex: Evidence for a Pronounced Multi-Facetted Metabolic Component in Rett Syndrome

**DOI:** 10.3390/cells10092494

**Published:** 2021-09-21

**Authors:** Gocha Golubiani, Vincenzo Lagani, Revaz Solomonia, Michael Müller

**Affiliations:** 1 Institut für Neuro- und Sinnesphysiologie, Zentrum Physiologie und Pathophysiologie, Universitätsmedizin Göttingen, Georg-August-Universität Göttingen, D-37130 Göttingen, Germany; gocha.golubiani.1@iliauni.edu.ge; 2 Institute of Chemical Biology, Ilia State University, 0162 Tbilisi, Georgia; vincenzo.lagani@iliauni.edu.ge (V.L.); revaz_solomonia@iliauni.edu.ge (R.S.)

**Keywords:** Rett syndrome, Mecp2, pathogenic mechanism, metabolism, carbohydrates, amino acids, mitochondria

## Abstract

Using unsupervised metabolomics, we defined the complex metabolic conditions in the cortex of a mouse model of Rett syndrome (RTT). RTT, which represents a cause of mental and cognitive disabilities in females, results in profound cognitive impairment with autistic features, motor disabilities, seizures, gastrointestinal problems, and cardiorespiratory irregularities. Typical RTT originates from mutations in the X-chromosomal methyl-CpG-binding-protein-2 (*Mecp2*) gene, which encodes a transcriptional modulator. It then causes a deregulation of several target genes and metabolic alterations in the nervous system and peripheral organs. We identified 101 significantly deregulated metabolites in the Mecp2-deficient cortex of adult male mice; 68 were increased and 33 were decreased compared to wildtypes. Pathway analysis identified 31 mostly upregulated metabolic pathways, in particular carbohydrate and amino acid metabolism, key metabolic mitochondrial/extramitochondrial pathways, and lipid metabolism. In contrast, neurotransmitter-signaling is dampened. This metabolic fingerprint of the Mecp2-deficient cortex of severely symptomatic mice provides further mechanistic insights into the complex RTT pathogenesis. The deregulated pathways that were identified—in particular the markedly affected amino acid and carbohydrate metabolism—confirm a complex and multifaceted metabolic component in RTT, which in turn signifies putative therapeutic targets. Furthermore, the deregulated key metabolites provide a choice of potential biomarkers for a more detailed rating of disease severity and disease progression.

## 1. Introduction

Rett syndrome (RTT) is among the leading causes of a severe cognitive impairment in females. It represents a neurodevelopmental disorder of monogenic cause, while giving rise to a complex clinical manifestation with a broad spectrum of symptoms, the severity of which may vary markedly among individuals [1,2]. The majority of patients affected by RTT carry mutations in their *MECP2* (methyl-CpG-binding-protein-2) gene [3], which is located on the long arm of the X-chromosome and encodes the transcriptional modulator MeCP2. In addition to this specific type of *MECP2* mutation, it is the X-chromosomal location, together with its resulting random X-mosaicism, that underlies the interindividual heterogeneity of symptomatic severities [4]. Typical symptoms that manifest in mostly female patients, after an apparently normal initial development, include: cognitive impairment with features of autism, susceptibility to seizures, motor dysfunction with improper posture, cardiac and gastrointestinal problems, and a highly distorted breathing pattern [5,6,7].

Based on these symptoms, RTT represents a mostly but not exclusively neurological disorder. More than three decades ago, first indications were obtained that RTT may also involve a mitochondrial component [8,9], and it is now becoming more evident that RTT is associated with a spectrum of metabolic alterations. Blood serum and cerebral fluid samples revealed elevated levels of lactate and pyruvate [10,11] in some patients. Both the upregulation of the glucose transporter SLC2A4 observed in the Mecp2-deficient mouse hippocampus [12] and the lower blood glucose levels in these mice [13] suggest an altered carbohydrate metabolism in RTT [14]. Furthermore, alterations in cholesterol homeostasis and distorted sphingolipid metabolism were detected in patient blood samples [15,16]. Similarly, the brains of Mecp2-deficient mice showed changes in phospholipid metabolism [17].

Recent multi-omics analyses on patient blood samples identified alterations in mitochondrial DNA [18], and further mutations in genomic DNA that are relevant for mitochondrial and redox-regulatory functions. In the liver and skeletal muscles of male Mecp2-deficient mice, indications of disturbed utilization of mitochondrial substrate were obtained, pointing to potential disturbances in the TCA cycle [19]. In the brains of *Mecp2*-mutant mice, mitochondrial impairment is indicated by altered mitochondrial activities, increased O_2_ consumption, and exaggerated ROS generation [20,21,22,23,24]. Furthermore, the oxidative stress that is inherent to RTT [25] increases various oxidative stress markers in patient fibroblasts and in blood samples [26,27,28,29].

In terms of cellular signaling, the levels of biogenic amines, substance P, and nerve-growth factors are modified in spinal fluid [30,31,32,33]. Moreover, various neurotransmitters such as acetylcholine and glutamate are markedly affected in RTT [17,34,35].

In view of the constantly high energy demand, the undisturbed function of neural tissue is particularly prone to such metabolic disturbances and synaptic alterations. Metabolomics is the ideal tool to determine the full extent of these changes. Detailed and quantitative information is provided on hundreds of small molecule substrates, intermediates, and products involved in cellular metabolism (for review see: [36]). This defines a unique fingerprint resembling the precise metabolic situation at the exact moment when the tissue was collected. Without doubt, the metabolic signature obtained is much more closely related to the precise phenotypic conditions than the proteomic or transcriptional datasets.

Metabolomic studies in RTT are currently in their early stages but bear the potential to provide global insights into pathogenic mechanisms. Recently, the suitability of this approach was demonstrated in a first metabolomics study on RTT patient-derived blood samples [37], and the characterization of the gut microbiome and metabolome in RTT patients [38]. An earlier pilot metabolic screening in male *Mecp2*-null (*Mecp2^−/y^*) mice analyzed full brain extracts by means of high-resolution magnetic resonance spectroscopy. Among the reported changes were a reduced choline phospholipid turnover, increased glutamine/glutamate ratios, and potential alterations in osmoregulation [17].

To obtain an unprecedented view of brain metabolism in a mouse model of RTT, we performed an untargeted metabolomics screening by comparing the cortices of adult male wildtype (WT) and male Mecp2-deficient (*Mecp2^−/y^*) mice. To the best of our knowledge, this represents a first-time detailed metabolomic characterization of the Mecp2-deficient cortex. We chose this specific brain region because it provides sufficient quantities of tissue, shows the highest mitochondrial ROS release, is markedly affected in RTT, and is characterized by a very tight coupling of neural function and metabolic conditions [39,40].

Having successfully detected more than 250 defined metabolites, we defined a steady-state metabolic signature of the severe disease state on postnatal day p50 in *Mecp2^−/y^* mice. We obtained clear evidence of various aspects of disturbed cellular metabolism and mitochondrial function, multiple distortions in cortical neurotransmitter levels and cellular signaling, and a broadly affected amino-acid metabolism. Because the metabolome is intimately linked to phenotypic conditions, this cortex-specific holistic view will be helpful in more precisely distinguishing between primary defects and secondary alterations provoked by the adverse disease-related conditions. In addition, it will initiate further areas of research, identify potential specific biomarkers for RTT diagnostics, and point to novel therapeutic concepts.

## 2. Materials and Methods

### 2.1. Mouse Model and Tissue Isolation

The current study was performed on the *Mecp2* knockout mouse model [B6.129P2(C)-Mecp2^tm1.1Bird^] [41]. We focused on the severe disease stage on postnatal day p50 and chose hemizygous male (*Mecp2^−/y^*) mice to ensure clearly defined genetic conditions with a total absence of Mecp2. The cortices of male WT and *Mecp2^−/y^* mice were processed individually (n = 6 each), phenotypic parameters were determined for each mouse, and the blood was analyzed during dissection (Table 1). The mice were decapitated under deep ether anesthesia, and cortices were isolated, flash-frozen in liquid N_2_ for rapid quenching, and cryopreserved at −80°C. Both the breeding of *Mecp2*-mutant mice, and all mouse tissue analyses and procedures, complied with the European and German animal welfare guidelines and were authorized by the Office of Animal Welfare of the University Medical Center Göttingen and by the Lower Saxony State Office for Consumer Protection and Food Safety (file number G16/2177).

### 2.2. Metabolomic Analyses

The comprehensive metabolomic analyses and the subsequent initial bioinformatics were conducted by a validated service provider (MetaSysX, Potsdam, Germany). In accordance with their validated protocols, the frozen tissue (~100 mg/sample) was ground, extracted in a single-step procedure (modified from [42]), and its volume adjusted to equal amounts of material. Polar/semi-polar primary and secondary metabolites were identified by UPLC-MS measurements using a Waters ACQUITY Reversed Phase Ultra Performance Liquid Chromatography (RP-UPLC), coupled to a Thermo-Fisher Exactive mass spectrometer (Orbitrap mass analyzer, electrospray ionization source). Chromatograms were acquired in full scan MS mode (100–1500 mass range) using C18 columns. Spectra were recorded in both positive and negative ionization modes. Primary metabolites were identified by GC-MS measurements. These were performed using an Agilent Technologies GC coupled to a mass spectrometer (Leco Pegasus HT), consisting of an electron impact ionization source and a time-of-flight mass analyzer. The spectra obtained were aligned and filtered. The extracted peaks were then annotated based on the MetaSysX database (UPLC-MS and GC-MC data) and the Fiehn Library (GC-MS data). During the annotation and identification of the respective metabolites, the sample data generated on the different platforms was normalized to their respective group medians to obtain normalized intensities.

### 2.3. Bioinformatic Analyses

All measurements were log-transformed to stabilize variance. Missing values were set to the average of their respective measurements. Differentially regulated metabolites were identified using the moderated *t*-test analysis implemented in the R package Limma [43], and upregulated and downregulated KEGG metabolism pathways were identified with the ROAST rotation-based test [44]. ROAST transforms Limma *t*-statistics into their equivalent z-scores from a standard normal distribution. A single deregulation statistic for each pathway is computed by taking the average of the z-scores from the metabolites belonging to this pathway. A *p*-value assessing the deregulation of each pathway is then obtained through a rotation test, a Monte Carlo technique that is more suitable for small sample sizes than permutation. Basically, ROAST identifies metabolites with a z-score larger than the square root of two (in absolute value) as those metabolites that contribute most to the up- or downregulation of a given pathway. Only uniquely identified metabolites were considered during pathway analysis, and pathways with less than three identified metabolites were discarded. All *p*-values were adjusted for false discovery rate [45], and the differential expression and ROAST analyses were performed with the BIOMEX software [46].

## 3. Results

**Figure 1:**
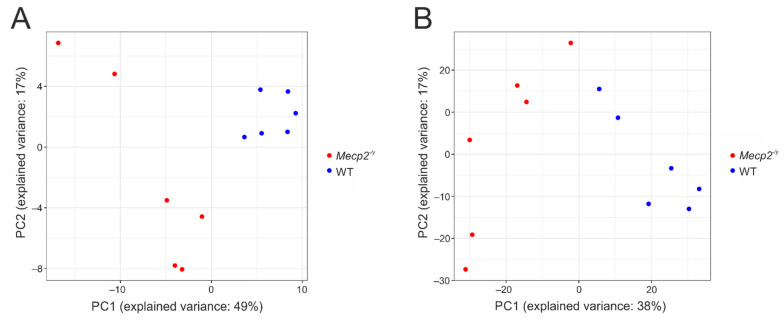
Principal component analysis (PCA) plots for *Mecp2^−/y^* (red dots) and WT (blue dots) cortices. PCA is computed only on the identified metabolites (**A**), and over all measurements (**B**). Percentage of explained variance is reported for both x (first component) and y (second component) axis.

We performed an untargeted metabolome analysis of the isolated cortices of adult WT and *Mecp2^−/y^* mice. These two groups of mice clearly differed in their general phenotypic appearance, with *Mecp2^−/y^* mice being smaller, weighing less, and presenting a tendency of slightly higher hematocrit and slightly lower blood glucose levels (Table 1). Metabolomics detected a total of 4143 features (2037 hydrophilic features in positive mode, 2003 hydrophilic features in negative mode, and 101 GC mass traces). Of these, 283 unique features were annotated based on the metaSysX database (Supplementary Materials Table S1), 32 of which could be matched to more than one reference compound (coeluting compounds). Principal component analysis shows that the WT and *Mecp2^−/y^* samples tend to cluster within their respective groups, when only the identified metabolites are included in the analysis (Figure 1A), and when all measurements are considered (Figure 1B).

Marked metabolic differences were detected among WT and *Mecp2^−/y^* cortex, with a total number of 101 identified metabolites differing significantly among genotypes at an adjusted *p*-value < 0.05 (Table 2). In detail, 33 metabolites were significantly decreased in the *Mecp2^−/y^* cortex, whereas the remaining 68 were significantly increased compared to WT. These differentially regulated metabolites represent compounds of different chemical nature, including amino acids, peptides, neurotransmitters, lipids, nucleosides/nucleotides, and carbohydrates. The five most severely downregulated (log2 fold changes) metabolites in *Mecp2^−/y^* cortex we identified are cysteinylglycine, L-homocysteine, L-glutamyl-L-glutamine, gamma-glutamyl-tyrosine, and 3-methoxytyramine. The five most severely upregulated metabolites are sucrose, D-glucose 6-phosphate, D-fructose 1,6-bisphospate, glyceraldehyde 3-phosphate, and rutin (see Table 2).

**Figure 2:**
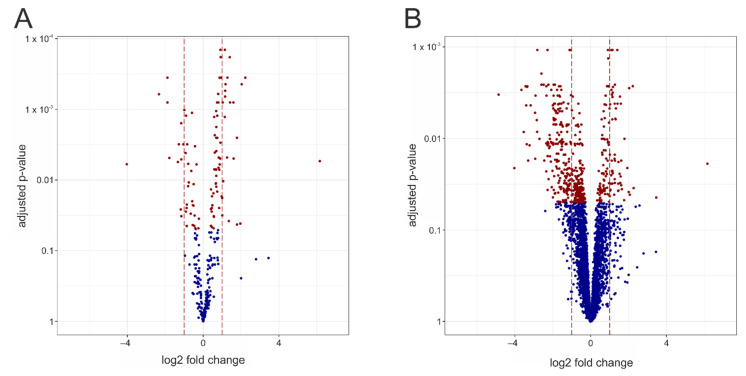
Volcano plots representing deregulated measurements between the *Mecp2^−/y^* and WT cortex. Each dot represents one known metabolite. The x-axis represents the log2 fold changes between *Mecp2^−/y^* and WT, and the y-axis the -log10 transformed adjusted *p*-values (as computed by a moderated *t*-test). Red dots correspond to those measurements with an adjusted *p*-value of less than 0.05, whereas blue dots indicate measurements that are not significantly deregulated. The brown vertical lines indicate the ±1 log2 fold change, corresponding to metabolites being present at double (+1) or half the level (−1) in the *Mecp2^−/y^* cortex. Volcano plots are shown for the identified metabolites (**A**) and for all measurements (**B**).

A volcano plot is used to visualize the results of the deregulation analysis and the complex genotype-related differences, i.e., the number of deregulated identified metabolites and their extent of alteration (Figure 2A). For unequivocal identification, red indicates significantly changed metabolites (FDR ≤ 0.05), whereas the non-significantly affected metabolites are shown in blue. A corresponding plot over all the measurements obtained is shown in Figure 2B.

**Figure 3:**
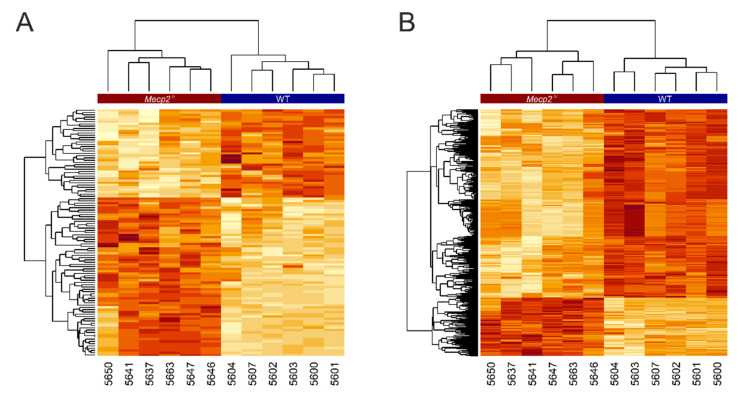
Heatmaps representing the concentration values of all deregulated identified/non-identified metabolites. Dark red indicates higher values and white lower values. Measurements are arranged in rows and the tissue samples are represented in columns. The *Mecp2^−/y^* and WT samples are labeled at the top of the heatmap by a red and blue banner, respectively. Dendrograms are included showing the clustering of both measurements and unique sample identifiers (individual mouse numbers). The heatmaps were computed for the identified metabolites (**A**) and for all measurements (**B**).

**Figure 4:**
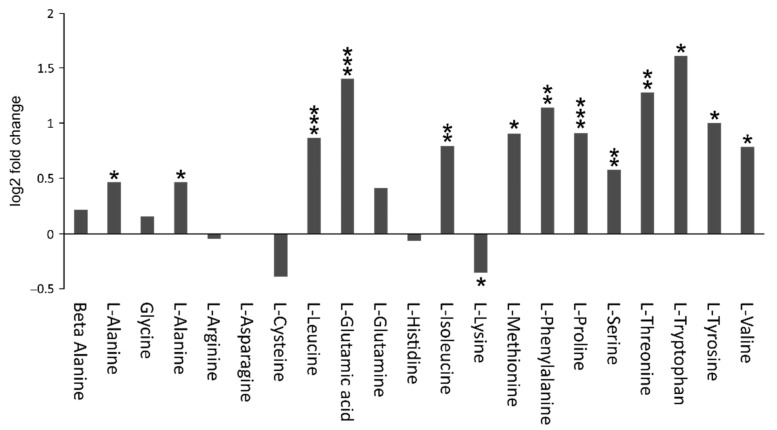
Several amino acids show clearly different levels in the *Mecp2^−/y^* and WT cortex. The majority were detected at higher levels in *Mecp2^−/y^* mice. Only lysine was decreased compared to WT conditions. Plotted are the log2 fold changes of the respective compounds. Asterisks indicate significant changes compared to WT (* *p* < 0.05, ** *p* < 0.01, *** *p* < 0.001).

The concentration values of all deregulated identified metabolites are illustrated in a heatmap (Figure 3A); a similar heatmap of all deregulated measurements was also generated (Figure 3B). These heatmaps reveal a very clear genotype-dependent clustering of the analyzed tissue samples. Furthermore, a distinct pattern of up- and downregulated groups of metabolites is evident for WT and the *Mecp2^−/y^* cortex. The volcano plots and heatmaps clearly indicate significant and drastic differences between the metabolomes of the *Mecp2^−/y^* and WT mouse cortex. In particular, this includes several amino acids, most of which were upregulated in the *Mecp2^−/y^* cortex (Figure 4). Lysine was the only amino acid showing a significantly decreased level compared to WT conditions. Furthermore, there was a relatively large number of metabolites yet to be identified. Some of these are highly discriminant between WT and *Mepc2^−/y^* mice, and may thus qualify as potential biomarkers for disease progression or symptom severity.

**Figure 5:**
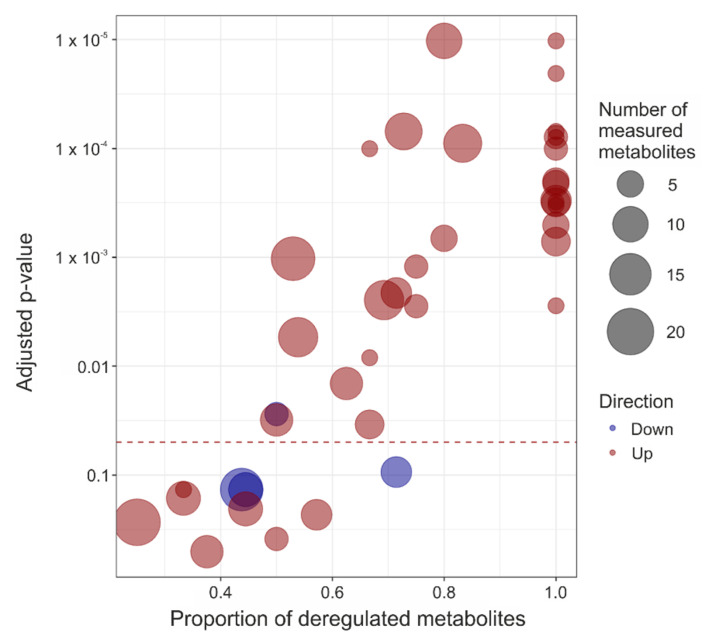
Graphical representation of the pathway enrichment results. Each dot represents a pathway, for which the x-axis represents the proportion of deregulated metabolites and the y-axis the adjusted *p*-value. The size of each dot is proportional to the number of identified metabolites present in the respective pathway, and the dot color indicates pathway up (red) or down (blue) regulation.

The high number of the significantly altered metabolites implies that several cellular pathways should be affected in the symptomatic *Mecp2^−/y^* mice. Therefore, we carried out a deregulation analysis at the pathway level, taking into consideration all mouse pathways relating to metabolism and included in the Kyoto Encyclopedia of Genes and Genomes (KEGG) [see: https://pubmed.ncbi.nlm.nih.gov/10592173/; accessed 16 July 2021]. In total, 41 metabolic pathways contained at least three identified metabolites, and 31 of these pathways were affected significantly in the *Mecp2^−/y^* cortex (FDR ≤ 0.05, Figure 5, Table 3). These deregulated pathways are associated, in particular, with carbohydrate and amino acid metabolism. However, general energy metabolism, lipid metabolism, and metabolism of cofactors and vitamins were modified to some degree (Table 3). The majority (30 of 31) of significantly deregulated pathways were upregulated in the *Mecp2^−/y^* cortex, compared to WT. This is obvious from the predominance of red color shades in the graphical representation of the pathway-enrichment analysis (Figure 5). Only glycerophospholipid metabolism was downregulated significantly in *Mecp2^−/y^* cortex. The alterations in nucleotide (pyrimidine, purine) metabolism and lysine degradation, in addition to histidine, tyrosine, tryptophan, beta-alanine metabolism, and glutathione metabolism did not reach the level of significance (FDR > 0.05). Neither vitamin B6, pantothenate, nor CoA biosynthesis differed significantly among the *Mecp2^−/y^* and WT cortex (Table 3).

**Figure 6:**
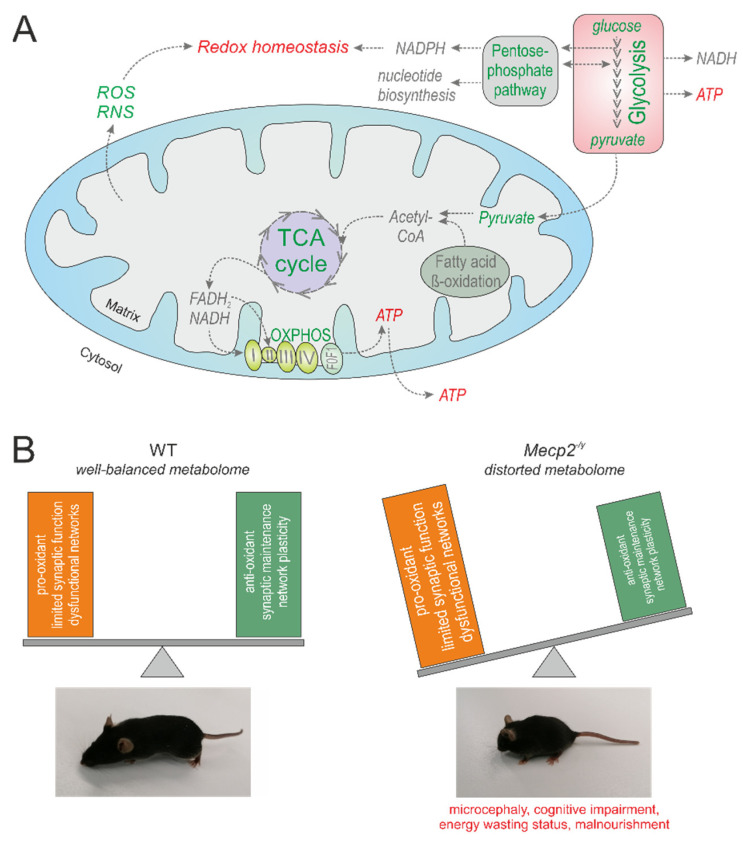
Multi-facetted metabolic derangements in the Mecp2-deficient mouse cortex. The central biochemical pathways that have been identified to be significantly altered by our unsupervised metabolomics analysis are outlined. Green font indicates upregulation, and red the downregulation and disturbance (**A**). It can be assumed that the entity of affected metabolites contributes to the characteristic features of RTT, which have been characterized in detail in numerous earlier studies. For example, this may include provoking a cellular redox imbalance with pro-oxidant conditions, facilitating conditions that limit synaptic function and network plasticity, and promoting an energy wasting status (**B**).

Because the significantly deregulated pathways involve various cellular activities, it is becoming clear that the cortex of *Mecp2^−/y^* mice suffers from multiple aspects of abnormal metabolism. This can be expected to considerably affect brain functioning. A detailed discussion of these changes is provided in the following section and a graphical summary of the deranged metabolism is presented in Figure 6.

## 4. Discussion

Our data convincingly indicates distinctive and significant differences between the metabolomes of the *Mecp2^−/y^* and WT mouse cortex. These differences were identified in mice kept under identical conditions and receiving an identical diet. As far as we know, this is the first study of its kind to specifically focus on whole-metabolome differences among the brain tissue of a RTT mouse model and WT mice. Previous studies of brain metabolite alterations in postmortem patient tissue samples or of *Mecp2*-mutant mice were ad hoc and targeted to a specific group of molecules, mainly neurotransmitters and their breakdown products (see below). Only one untargeted metabolome study has been conducted to date, focusing on the blood plasma of RTT patients and control subjects [37]. It must be considered that we studied the most severe conditions arising in the adult male, hemizygous *Mecp2^−/y^* mouse model. Whether the X-chromosomal mosaicism present in female heterozygous mice may be associated with less pronounced metabolic derangements remains to the clarified.

RTT is a neurodevelopmental disorder, caused by mutations of the transcriptional modulator MeCP2 [3], and available data confirms its association with changes in the expression of myriad genes (e.g., [47,48]). One of the major challenges not only for RTT, but also for other neuropathologies, is to determine which of these changes contribute causally to the pathological conditions, which are compensatory, and which are non-contributory. We discuss our findings and other available information within this context.

### 4.1. Carbohydrate Metabolism

Considering the changes observed in glycolysis, we suggest that glucose metabolism is intensified in the cortex of *Mepc2^−/y^* mice because the first two components of glycolysis, namely glucose and glucose-6-phosphate, are elevated in this brain region. In addition, levels of fructose 1,6-bisphosphate, glyceraldehyde 3-phosphate, and pyruvic acid were significantly increased. In view of an intensified glycolytic rate, an augmented glucose uptake into the brain can also be assumed. This may explain our earlier findings on the upregulation of the glucose transporter SLC2A4 [12] and the lowered blood glucose level in *Mecp2^−/y^* mice [13], which is also evident as a trend in the current cohort of mice (Table 1).

Moreover, the pentose phosphate pathway is upregulated in the *Mecp2^−/y^* cortex (Table 3). This pathway is a source of nicotinamide adenine dinucleotide phosphate (NADPH), which is then fed into reductive biosynthesis reactions and contributes to cellular redox homeostasis. Therefore, its upregulation may be considered compensatory in providing the oxidatively stressed cortex with proper amounts of reducing equivalents.

It was recently shown that erythritol, a reduced form of the monosaccharide erythrose, and which, in its phosphorylated form, is an intermediate of the reductive pentose-phosphate pathway, may be formed endogenously from glucose via the pentose phosphate pathway [49]. The level of erythritol is increased in the *Mecp2^−/y^* mouse cortex, which may be a consequence of the intensified carbohydrate metabolism. Interestingly, increased erythritol contents have also been detected in the brains of mentally ill patients [50]. Because severe cognitive impairment is among the characteristics of RTT, the relevance of the increased erythritol level should be clarified by further research.

Within the tricarboxylic acid (TCA) cycle, seven metabolites are upregulated in *Mecp2^−/y^* cortex. Five of these, namely, succinic acid, L-malic acid, alpha-ketoglutaric acid, citric acid, and thiamine pyrophosphate, a cofactor in the pyruvate decarboxylation reaction by the pyruvate dehydrogenase complex, are substantially increased (Table 3). This clearly confirms the dysregulation of this pathway in the *Mecp2^−/y^* mouse cortex.

To the best of our knowledge, our metabolomics data is the first direct evidence for changes in the TCA cycle in the *Mecp2^−/y^* brain. The TCA cycle is clearly among the most severely affected central pathways with seven identified and upregulated metabolites. In patients with RTT, the cerebrospinal fluid (CSF) was analyzed for lactate, pyruvate, and citric acid cycle intermediates [10]. Of the citric acid cycle metabolites, only alpha-ketoglutarate and malate were significantly elevated in these patients compared to controls. Because the CSF metabolome is a reflection of the brain metabolome, this report also indicates indirectly increased brain tissue levels of citric acid cycle metabolites.

The TCA cycle is of central importance for various downstream biochemical pathways. One of its main functions is to release energy through the oxidation of acetyl-CoA derived from carbohydrates, fats, and proteins, and to provide NADH / FADH_2_ for mitochondrial respiration. Accordingly, the TCA cycle is tightly regulated, and ATP acts as an allosteric inhibitor of pyruvate dehydrogenase and isocitrate dehydrogenase. It is well known that high demands for ATP increase the ADP/ATP ratio and AMP levels, thereby stimulating the regulatory enzymes of the TCA cycle (reviewed in [51]). Therefore, we cannot exclude the possibility that the observed increase in the TCA cycle metabolites is the result of an abnormal regulation of the cycle itself, and a compensatory attempt of the Mecp2-deficient organism. Increased activity of the TCA-enhanced glycolytic activity would increase the total amount of energy provided, while at the same time increasing the availability of reduced NADH to be fed into, e.g., the mitochondrial respiratory chain.

Recent research indicates an additional, new role of TCA cycle intermediates in signaling molecules controlling chromatin modifications, DNA methylation, hypoxic responses, and immunity (reviewed in [51]). Succinate stabilizes the transcription factor hypoxia-inducible factor (HIF)-1α in specific tumors and activated macrophages, stimulates dendritic cells via its receptor succinate receptor 1, and modifies proteins post-translationally (reviewed in [52]). Whether such extended succinate-mediated signaling also applies to Mecp2-deficient brains remains to be clarified, but it may shed light on our previous reports about modified hypoxic responses and brain-wide increased HIF-1α expression levels in *Mecp2^−/y^* mice [53,54].

### 4.2. General Energy Metabolism

The levels of ADP, AMP, and orthophosphate were dramatically increased in the cortex of *Mecp2^−/y^* mice. Neurons account for most (∼80–90%) of the energy demand of the brain [55]. Therefore, these differences can be expected to reflect changes mostly in neuronal energy metabolism. Neuronal activity requires highly intense expenditure and resupply of metabolic energy. ADP is a well-known effector of oxidative phosphorylation and is considered to be a proximal signal that coordinates metabolic responses to high energy demand [56]. AMP is considered an activator of glycolysis (see above). It facilitates the activation of 5′-AMP-dependent protein kinase (AMPK), which rapidly triggers the activation of 6-phosphofructo-1-kinase—the master regulator of the glycolytic pathway [57]. Thus, the detected increases in ADP and AMP may partially explain the intensified glycolysis and TCA cycle in the *Mecp2^−/y^* cortex.

Mitochondrial electron transport chain (ETC) activity cannot be rated in detail by metabolomics. This requires high resolution respirometric approaches [58]. Nevertheless, in view of the increased substrate levels, our pathway analyses identified the oxidative phosphorylation as upregulated (Table 3). The intensified TCA cycle and glycolysis translocate more substrates into the mitochondrial ETC (Figure 6A). This could be a means of compensating for the mitochondrial impairments and the inefficient mitochondrial respiration detected in RTT [21,22,23,24]. Moreover, the increased substrate levels themselves may provoke a dysregulation of the mitochondrial ETC. In support of these assumptions, increased energy expenditure and intensified ATP turnover were brought to light in neonatal hippocampal *Mecp2^−/y^* neurons [59], and a mitochondrial energy-wasting status has been proposed for RTT [60]. This may explain why decreased ATP levels were found in full brain-analyses on male and female *Mecp2*-mutant mice [21,61]. However, focusing on adult *Mecp2^−/y^* hippocampus, we did not observe decreased ATP levels in that specific brain region [53]. This emphasizes the need for more thorough region-specific studies of brain energetics in *Mecp2*-mutant mice. Only then can the full picture be obtained.

### 4.3. Amino Acid Metabolism

Amino acid metabolism is one of the most severely affected features in the *Mecp2^−/y^* mouse cortex, the majority of amino acids showing clearly increased levels compared to WT (Figure 4). To date, information on altered amino acid levels in RTT is scarce. In a single case report on postmortem brain tissue, a tendency of lowered levels of several amino acids was found in pallidum, putamen, caudatum, white matter, and thalamus [62]. However, these concomitant changes could not be observed in CSF [62]. A more recent study revealed in RTT patient-derived blood samples clear changes in amino acid levels, with an increase or trending increase in aspartate, glutamate, cysteine, glycine, and serine, and a decrease or trending decrease in arginine, histidine, and phenylalanine [37]. In contrast, an RTT mouse study on full brain extracts yielded only slight changes in amino acid levels (glutamine increased, GABA trend to decrease). This is perhaps due to the fact that this magnetic resonance spectroscopy analysis has only quantified a few metabolites, and the use of full brain extracts may have masked brain-region specific details [17].

In our study on the mouse cortex, the vast majority of amino acids were detected at increased levels. Hence, the often-stated condition of chronic undernutrition in RTT, which is also evident from the notably reduced body weights of the *Mecp2^−/y^* mice (Table 1), cannot account for these changes. Thirteen of the 20 proteinogenic amino acids (L-alanine, L-glutamic acid, L-leucine, L-isoleucine, L-lysine, L-methionine, L-phenylalanine, L-proline, L-serine, L-threonine, L-tryptophan, L-tyrosine, and L-valine) showed significantly elevated concentrations in the *Mecp2^−/y^* cortex (see also Table 1, Figure 4). A metabolomics study of RTT patient’s plasma revealed significant changes in four proteinogenic amino acids: aspartate and glutamate were upregulated, whereas arginine and histidine were downregulated [37]. In our study, only one of the four, L-glutamic acid, was detected as being significantly upregulated. The remaining three did not differ, which may be due to the different species (human vs. mouse) and specimen sources (cortex vs. blood plasma) of the studied metabolomes. The observed differences emphasize once more the importance of localized, tissue specific analyses of neuropathological conditions. The distinctive increase in more than half of proteinogenic amino acids does not only indicate an abnormal protein synthesis, but also changes in cellular pathways linked to individual or specific groups of amino acids. Indeed, earlier data indicates an impaired protein synthesis in various parts of the brain, including the cortex, at early pre-symptomatic stages of RTT [63].

The group of branched amino acids (BAAs) consists of L-leucine, L-isoleucine, and L-valine, and all three showed significant increases in concentration in the *Mecp2^−/y^* cortex. BAAs, in addition to protein synthesis, are involved in several important brain functions including nitrogen homeostasis and neurotransmitter cycling, and they can be utilized as energy substrates in the TCA cycle (reviewed in [64]). Changes in BAA concentrations are linked to neuropathological conditions. Incubation of cerebral cortex homogenates with L-leucine elicits oxidative stress by increasing thiobarbituric acid-reactive substances [65]. Exposure of cultured cortical astrocytes to BAAs alters cell morphology and cytoskeletal organization [66]. Accordingly, elevated BAA levels may contribute to the oxidative stress in RTT and to the intensified TCA cycle indicated by our data.

The list of deregulated amino acids also contains the non-proteinogenic amino acid L-homocysteine, which is considerably decreased in the *Mecp2^−/y^* cortex. L-homocysteine is a sulfur-containing amino acid. It derives from S-adenosylmethionine, an important source of methyl groups in methylation reactions, such as DNA methylation or catecholamine synthesis (reviewed in [67]). L-homocysteine is formed in the metabolism of methionine, which is upregulated in the *Mecp2^−/y^* cortex (see above). Accordingly, L-homocysteine should then also be upregulated. Yet, the opposite is true, which suggests other regulation pathways dampen its levels. Accordingly, this may result in a less pronounced methylation of target molecules. A potential cause may be the oxidative burden in RTT, which would force the oxidation of L-homocysteine. In keeping with the suspected sulfhydryl oxidation, L-cystin was increased in the *Mecp2^−/y^* cortex (Table 2).

The mechanistic cause of RTT is the functional disruption of MeCP2, which also acts as a transcriptional repressor (reviewed in [68]) by binding to methylated CA sites within long genes. Accordingly, in neurons lacking MeCP2, a decreased expression of long genes attenuates the RTT-associated cellular deficits [69]. These long genes represent a population of genes that are crucial for neuronal function and are expressed selectively in the brain [69]. It is intriguing to suggest that a decreased potency of DNA methylation, due to downregulated L-homocysteine, may be an attempt of Mecp2-deficient cells to balance to some extent unoccupied methylated DNA binding sites.

### 4.4. Dipeptides

The levels of 13 dipeptides differed significantly among the *Mecp2^−/y^* and WT cortex. These include L-glutamyl-L-glutamine, gamma-glutamyl-tyrosine, phenylalanyl-L-glutamic acid, cysteinyl-glycine, L-valyl-glycine, gamma-glutamyl-leucine, L-tryptophyl-L-glutamic acid, L-tyrosyl-glycine, L-tyrosyl-L-glutamine, L-prolyl-L-threonine, gamma-glutamyl-tryptophan, L-valyl-L-alanine, and phenylalanyl-L-threonine. The levels of five dipeptides (L-glutamyl-L-glutamine, gamma-glutamyl-tyrosine, cysteinyl-glycine, gamma-glutamyl-leucine, and gamma-glutamyl-tryptophan) were decreased and four of these are glutamate-containing peptides. The physiological functions of most of these differentially regulated dipeptides are characterized.

Gamma-glutamyl leucine levels are significantly decreased in the plasma of patients with major depressive disorder [70]. It was suggested that the low levels of gamma-glutamyl leucine reflect low glutathione turnover, which is the main protective cellular antioxidant and plays a pivotal role in oxidant/antioxidant balance [71,72]. Indeed, the cellular redox balance is seriously disrupted in the brain of RTT mice and patients [12,22,24,26,73,74].

Cysteinyl-glycine is supplied by astrocytes for the neuronal synthesis of glutathione [75]. Thus, the decreased level of this dipeptide may contribute to decreased glutathione synthesis and disrupt antioxidant capacity in the *Mecp2^−/y^* mouse cortex. Interestingly, of all the compounds identified in the *Mecp2^−/y^* cortex, cysteinyl-glycine is the most severely downregulated metabolite (log2 fold change of −4.03).

Histidine-containing dipeptides mediate cellular protection by detoxifying reactive carbonyls, which arise from oxidant-mediated tissue damage, in particular, the oxidation of sugars and polyunsaturated fatty acids (see [76]). In our study, histidine-dipeptides were not among the uniquely identified metabolites. However, one of the markedly decreased features in the *Mecp2^−/y^* cortex was annotated to three different reference compounds (L-carnosine, L-histidylalanine, and L-alanyl-L-histidine; see Supplemental Matrials Table S1), all of which are histidine-containing peptides capable of detoxifying carbonyls. Hence, the oxidative burden in RTT is likely to diminish each of these peptides.

### 4.5. Urea

This metabolite is clearly increased in the cortex of *Mecp2^−/y^* mice, and urea cycle disorders are associated with cognitive and motor deficits [77]. Urea is formed in the urea cycle by arginase mediated cleavage of arginine. The urea cycle activity is primarily localized in the liver, but also occurs in other cell types. In the brain, a partial urea cycle appears to function mainly to degrade the two amino acids citrulline and arginine [78]. Of the various urea cycle components, we identified the metabolites ornithine, citrulline, arginosuccinate, fumarate, and arginine, but only arginosuccinate was significantly upregulated (Table 2). The synthesis of carbamoyl phosphate is the rate-limiting step in the urea cycle. This specific metabolite was not detected/annotated in our analysis. From the mechanistic perspective, liver failure should be considered as a cause of the increased urea levels in the *Mecp2^−/y^* cortex, because *Mecp2* deletion in mice also results in a fatty liver [79]. It should be mentioned, however, that urea levels did not differ in the plasma of RTT patients and control subjects (see [37], Supplementary Materials Table S3 of this reference).

Urea levels are increased in the brains of patients with both Alzheimer´s [80] and Huntington´s disease (HD), including those with low-level HD neuropathology [81,82], and in the brain of a transgenic sheep model of HD [81]. RNA-Seq analysis also identified significantly increased levels of the urea transporter SLC14A1 in the striatum of these HD sheep [81]. The cerebral urea transporter is expressed mainly in astrocytes [83] and mediates the facilitated diffusion of urea [84]. Therefore, the increased SLC14A1 expression was considered a direct response to the elevated brain urea levels in HD [81].

### 4.6. Neurotransmitters

The neurotransmitters acetylcholine, dopamine, and serotonin are significantly downregulated in the cortex of *Mecp2^−/y^* mice. Furthermore, choline, which is both a precursor and a breakdown product of acetylcholine, and methoxytyramine, a dopamine metabolite, are decreased in the *Mecp2^−/y^* cortex. Initial studies of postmortem brains of RTT patients indicated decreased levels of dopamine in different regions, including the cortex [85,86,87]. This, however, was contradicted by another study [88], which claimed normal levels of dopamine and its metabolites in all brain regions. More recent analyses have targeted dopamine receptors and dopamine transporters both in humans and *Mecp2*-deficient mice [89]. Only marginal differences were found in the case of dopamine transporters. D2 dopamine receptors (D2R) were reduced in the striatum of RTT patients and hemizygous/heterozygous Mecp2-deficient mice compared to controls. Because the cortex was not included in this study [89], it remains debatable whether the reduced dopamine and methoxytyramine levels observed during our research may be linked to an altered dopamine receptor expression.

Early studies of postmortem brains of RTT patients also indicated a downregulation of the serotonergic system. In parallel with low levels of serotonin, a decreased amount of the rate-limiting enzyme of its biosynthesis tryptophan hydroxylase-2 was reported [62]. A cell-autonomous decrease in brain serotonin levels was caused by specifically eliminating the *Mecp2* gene in serotonergic neurons. This suggests that the decreased serotonin content is not due to a Mecp2 dysfunction in other cell populations [30], and thus may contribute directly to the onset or the progression of the disease. A more recent study showed that fluoxetine rescues motor coordination in *Mecp2* heterozygous mice through its ability to enhance the brain serotonergic system, which suggests that drugs stabilizing 5-HT neurotransmission may ameliorate the motor symptoms in RTT [90,91].

To the best of our knowledge, our study is the first to demonstrate a decreased level of acetylcholine in the brain of *Mecp2*-mutant mice. Numerous studies have focused on the levels of choline, expression of enzymes involved in acetylcholine metabolism, vesicular acetylcholine transporters, and acetylcholine receptors in postmortem RTT patient tissue or mouse models of RTT [34,92,93,94,95]. All of these indicate a downregulation of cholinergic system functioning. Recently, *Mecp2* was selectively deleted from cholinergic neurons in mice [96], causing a selective impairment of recognition memory and profound alterations in baseline firing of L5/6 neurons. These behavioral and electrophysiological deficits were rescued by inhibiting ACh breakdown [96]. Together with our results, this indicates that decreased ACh levels are not a side effect of Mecp2 disruption but actually contribute to disease development.

Our data also indicates increased amounts of glutamate and glutamine in the *Mecp2^−/y^* cortex. Glutamate serves various cellular functions. It is a canonical amino acid used for protein synthesis, acts as a neurotransmitter, and is a precursor of the neurotransmitter gamma-aminobutyric acid (GABA). Because GABA was not identified unequivocally in our analysis, we cannot judge its differences in the *Mecp2^−/y^* cortex. Glutamate exists as free amino acid inside cells, packed as a neurotransmitter in synaptic vesicles, or as a released neurotransmitter in the synaptic cleft and extracellular space. In our experiments, we detected global glutamate amounts and it is impossible to assess which particular fraction was increased. In astrocytes, released glutamate is converted to glutamine and supplied to neurons where it is re-converted to glutamate. Thus glutamine, together with its canonical amino acid function, is both a precursor of glutamate and a product of its metabolism.

To date, two studies have addressed glutamate levels in *Mecp2*-mutant and WT mice [17,97]. Whereas whole brain analysis did not reveal genotype-related differences in glutamate content [17], the brain region specific analyses, which also included the motor cortex, were more successful and detected lowered glutamate levels only in the hippocampus of *Mecp2^−/y^* mice [97]. Because the balance between synaptic excitation and inhibition is impaired in Mecp2-deficient mice [98,99], the increased levels of glutamate and glutamine detected could contribute to these changes. Yet, in mouse models of RTT, the cortical circuits are rendered hypoactive, showing a decreased excitation and an increased inhibition [98]. Therefore, it remains to be clarified how exactly the increased glutamate and glutamine contents contribute to these conditions.

The specific deletion of *Mecp2* in a subset of GABAergic forebrain interneurons replicates several typical features of RTT: Mecp2-deficient GABAergic neurons present lowered levels of glutamic acid decarboxylase 1 and 2, and a diminished GABA immunoreactivity [100]. Based on this report, we propose that the increased cortical levels of glutamate are not sufficient to rescue the lower GABA levels in *Mepc2^−/y^* mice.

In our metabolomic analyses, significantly decreased cAMP levels were not found in *Mecp2^−/y^* cortex, but 5′ AMP levels were significantly increased. Hence, a partly disturbed cAMP-homeostasis may be assumed, which may contribute further to the disturbed synaptic signaling in *Mecp2*-deficient brains.

### 4.7. Lipid Metabolism

The quaternary ammonium compound (3-carboxypropyl) trimethylammonium (butyrobetaine) is significantly decreased in the *Mecp2^−/y^* cortex. In mammals, the precursor butyrobetaine is converted to carnitine in a reaction catalyzed by gamma-butyrobetaine dioxygenase (reviewed in [101]). The amount of carnitine also tends to be less in the *Mecp2^−/y^* cortex, but the difference is not statistically significant. Carnitine is essential for the translocation of long-chain fatty acids into mitochondria for β-oxidation [101]. It was recently proved that up to 20% of the total brain’s energy is provided by the mitochondrial oxidation of fatty acids, which almost exclusively occurs in astrocytes (reviewed in [102]). We therefore speculate that the decreased levels of butyrobetaine may contribute to the energy deficiency in the *Mecp2^−/y^* cortex. Alterations of the carnitine cycle were also uncovered in cardiac tissue of female *Mecp2^+/−^* mice, and were linked to an upregulated carnitine palmitoyltransferase 1 A/B and carnitine acylcarnitine translocase [103].

The level of arachidonic acid [(5Z,8Z,11Z,14Z)-Icosatetra-5,8,11,14-enoic acid] is also significantly increased in the *Mecp2^−/y^* mouse cortex. Arachidonic acid is a target of free-radical catalyzed reactions, which generate isoprostanes. Therefore, isoprostanes are considered lipid peroxidation products, and their amounts are increased in the brains of *Mecp2*-null mice and in patient blood samples [26,27]. Isoprostanes are not among our identified metabolites. Nevertheless, based on the oxidative stress in RTT, arachidonic acid could become increasingly oxidized and more lipid peroxidation products should also accumulate in the *Mecp2^−/y^* cortex, as was demonstrated for full brain extracts of Mecp2-deficient mice [26]. Furthermore, as arachidonic acid is pro-inflammatory, it may contribute to the pro-inflammatory conditions (OxInflammation) in RTT [104].

Altered cholesterol metabolism was also reported in the brain and liver of *Mecp2^−/y^* mice and plasma cholesterol levels were increased [105]. Consistent with these findings, our study confirms significantly increased cholesterol levels in the *Mecp2^−/y^* cortex.

### 4.8. Markers of Oxidative Stress

Our analyses indicate various deregulated metabolites, which reveal the impact of the RTT-associated oxidative stress on the cortical tissue. This includes the above-mentioned lowered levels of L-homocystein and cysteinly-glycine, and the increased contents of L-cystin, branched amino acids, and rutin. Rutin, a flavonoid glycoside (found in e.g., buckwheat and apples), has a strong antioxidant property. It is metabolized by the gut microbiome [106]. In RTT, the gut microbiome, depending on clinical severity score, has been reported to shift towards a less diverse and pro-inflammatory composition [38,107]. This may result in an altered bioavailability of non-metabolized rutin in *Mecp2^−/y^* mice. Another response to the oxidative stress may be the increased supply of reduced reduction equivalents by the intensified glycolysis, pentose phosphate pathway, and TCA cycle, as summarized graphically in Figure 6A. Moreover, L-dihydroascorbic acid, an oxidized form of ascorbic acid, was increased in the *Mecp2^−/y^* cortex. The content of ascorbic acid itself was not affected. Nevertheless, the accumulation of its oxidized form in the *Mecp2^−/y^* cortex appears to be another consequence of the oxidative burden. In accordance with this concept, earlier analyses of postmortem brain tissue of an RTT patient detected a reduced ascorbic acid content in various parts of the brain, including the cortex [108].

### 4.9. Concluding Remarks

Our unsupervised metabolomic analyses identified a multitude of affected metabolites in the cortex of *Mecp2^−/y^* mice. This clearly confirms that RTT involves a highly complex and critical metabolic component that markedly affects several of the most central metabolic pathways. Accordingly, these central pathways—in particular carbohydrate and amino acid metabolism—should be considered when evaluating therapeutic approaches in RTT and/or when developing further treatment concepts. The complex metabolic distortions are clearly not restricted to the cortex but bear a systemic relevance. Thus, the intriguing question arises regarding the extent to which the remaining brain regions and peripheral organs may be affected. Due to the low quantities of tissue required, metabolomics can be extended to other brain regions or organs of individual mice. However, an integrated response of the particular tissue is obtained, which contains the metabolic signatures of all cell types present. This also applies to the cortex studied here, which is a complex and highly specialized brain region. Accordingly, cell-type or even neuronal-subtype-specific metabolic alterations cannot be identified, even though this information would be of tremendous interest for a more comprehensive understanding of RTT pathogenesis.

Nevertheless, metabolomics does provide a multitude of valuable details on the phenotypic conditions at the specific time point at which a tissue is collected. Here, we present a pilot study on the *Mecp2^−/y^* mouse cortex during which a total of 283 metabolites were successfully identified. In addition, as indicated by the volcano plots and heatmaps, there are a large number of highly discriminant features present in the WT and *Mecp2^−/y^* cortex that remain to be identified. Accordingly, with the further development of metabolomics and the constantly growing capacity of reference databases, even more detailed metabolomic fingerprints can be expected in future studies. The remaining challenge will then be to identify those few metabolites that uniquely characterize RTT from the multiple deregulated metabolites, several of which are involved in a spectrum of pathological conditions. In a clinical application, this may specify the detailed conditions of individual patients, which is of ultimate relevance regarding the design and evaluation of personalized therapy options. The field of metabolomics will also become indispensable for the further deciphering of pathogenic details and the characterization of the different severities, disease stages, and variants of RTT.

## Figures and Tables

**Table 1:** Phenotypic features of the analyzed WT and *Mecp2^−/y^* mice, including the respective means ± standard deviations. Genotypic comparison was performed in a two-tailed two-sided unpaired *t*-test (see *p*-values).

**WT Mice (*n* = 6)**				
Identifier	Body size [cm]	Body weight [g]	Blood glucose [mg/dL]	Hematocrit
	8.23 ± 0.21	21.60 ± 1.06	242.50 ± 30.57	43.58 ± 2.05
#5600	8.2	22.7	211	46.3
#5601	8.6	22.6	247	45.0
#5602	8.1	20.0	243	41.3
#5603	8.4	22.5	235	45.3
#5604	8.0	21.2	215	41.0
#5607	8.1	20.6	304	42.6
***Mecp2^−/y^* Mice (*n* = 6)**				
	7.17 ± 0.69 (*p* = 0.0078)	13.78 ± 3.70 (*p* = 0.0011)	200.00 ± 50.65 (*p* = 0.1393)	46.4 ± 2.46 (*p* = 0.0777)
#5637	6.9	11.5	177	50.0
#5641	6.5	11.4	147	47.0
#5646	8.3	20.5	306	47.7
#5647	7.9	17.0	208	46.7
#5650	6.9	12.1	185	42.0
#5663	6.5	10.2	177	45.0

**Table 2:** List of the identified 101 metabolites that differ significantly between *Mecp2^−/y^* and WT cortices (FDR ≤ 0.05). For each metabolite the common name, log2 fold change, raw *p*-value, and adjusted *p*-value are reported. Only those metabolites are listed that could be identified (annotated) unequivocally in the database.

Metabolite	Log Fold Change	Average Expression	*p*-Value	Adjusted *p*-Value
L-Proline	0.910	19.269	1.02 × 10^−6^	0.00014
L-Glutamic acid	1.406	19.899	2.04 × 10^−6^	0.00018
N-Acetylglutamic acid	1.146	13.495	7.33 × 10^−7^	0.00014
Adenosine monophosphate	0.925	20.923	3.23 × 10^−6^	0.00018
Adenosine diphosphate	0.925	20.923	3.23 × 10^−6^	0.00018
L-Threonine	1.281	18.628	9.24 × 10^−6^	0.00036
L-Leucine	0.870	17.672	1.18 × 10^−5^	0.00036
D-Glucose 6-phosphate	2.226	12.018	1.23 × 10^−5^	0.00036
Citric acid	0.872	18.512	1.38 × 10^−5^	0.00036
Inositol 1-phosphate	0.959	13.174	1.45 × 10^−5^	0.00036
(S)-Methyl-3-hydroxybutanoate	0.935	16.364	1.51 × 10^−5^	0.00036
Putrescine	1.179	14.878	2.05 × 10^−5^	0.00044
D-Fructose 1,6-bisphosphate	2.035	12.807	2.18 × 10^−5^	0.00044
D-Fructose	1.167	15.985	2.89 × 10^−5^	0.00055
L-Valine	0.787	18.180	3.55 × 10^−5^	0.00060
L-Isoleucine	0.794	16.510	3.63 × 10^−5^	0.00060
L-Homocysteine	−2.332	19.231	3.89 × 10^−5^	0.00061
Inosine 5prime-monophosphate	1.158	15.513	4.43 × 10^−5^	0.00066
D-Glucose	1.418	14.891	5.85 × 10^−5^	0.00080
Urea	1.599	18.093	6.27 × 10^−5^	0.00080
L-Glutamyl-L-glutamine	−1.885	27.116	6.07 × 10^−5^	0.00080
5prime-Deoxy-5prime-(methylthio)adenosine	0.732	12.146	6.81 × 10^−5^	0.00080
(5Z,8Z,11Z,14Z)-Icosatetra-5,8,11,14-enoic acid	1.032	12.834	6.63 × 10^−5^	0.00080
L-Rhamnose	0.805	15.911	7.07 × 10^−5^	0.00080
S-Adenosyl methionine	−0.994	23.740	9.39 × 10^−5^	0.00102
L-Threonic acid	0.685	16.744	0.00010	0.00106
Xanthine	−0.590	24.310	0.00011	0.00112
Glycerol	0.743	19.067	0.00013	0.00123
Uracil	−0.903	21.188	0.00013	0.00123
L-Serine	0.580	20.901	0.00014	0.00128
L-Phenylalanine	1.143	16.688	0.00014	0.00128
Riboflavin	−1.161	20.071	0.00018	0.00157
Erythritol	0.696	13.022	0.00021	0.00178
Orthophosphate	0.612	20.971	0.00029	0.00232
L-Dehydroascorbic acid	0.691	21.847	0.00032	0.00252
alpha-Ketoglutaric acid	1.783	12.766	0.00033	0.00252
Succinic acid	0.743	17.429	0.00034	0.00252
L-Methionine	0.909	16.361	0.00041	0.00296
1-Methyl-4-Imidazoleacetic acid	−0.837	21.803	0.00044	0.00312
Dopamine	−1.285	21.788	0.00046	0.00312
Choline	−0.431	26.902	0.00051	0.00333
L-Malic acid	0.695	17.421	0.00060	0.00389
alpha-D-Glucose 1-phosphate	0.608	16.334	0.00065	0.00409
Acetylcholine	−0.927	23.071	0.00067	0.00415
Guanidineacetic acid	0.821	20.099	0.00080	0.00480
Cholesterol	1.261	19.067	0.00081	0.00480
gamma-Glutamyl-tyrosine	−1.785	19.524	0.00084	0.00487
L-Phenylalanyl-L-glutamic acid	0.898	18.323	0.00089	0.00497
L-Tryptophan	1.611	14.883	0.00090	0.00497
Sucrose	6.158	13.392	0.00101	0.00541
3-Methoxytyramine	−1.329	19.231	0.00106	0.00555
Hydroxymethylphosphonic acid	−0.787	23.679	0.00115	0.00590
Cysteinylglycine	−4.028	21.772	0.00118	0.00598
L-Cystine	0.874	11.928	0.00123	0.00601
L-Lysine	−0.351	24.204	0.00123	0.00601
Xylitol	0.809	14.890	0.00140	0.00671
L-Valylglycine	0.679	18.356	0.00151	0.00707
myo-Inositol 2-phosphate	0.595	12.668	0.00152	0.00707
Quinic acid	0.856	11.418	0.00157	0.00714
Pantothenic acid	0.685	13.364	0.00159	0.00714
gamma-Glutamyl-leucine	−0.625	20.675	0.00168	0.00745
L-Homoserine	0.690	13.175	0.00201	0.00876
L-Tryptophyl-L-glutamic acid	1.067	17.063	0.00243	0.01042
O-Acetyl-L-homoserine	−0.758	25.057	0.00256	0.01082
2-Hydroxypyridine	0.676	18.513	0.00267	0.01111
L-Tyrosylglycine	0.669	17.228	0.00276	0.01131
Cytidine	−0.498	24.940	0.00283	0.01144
3-Ureidopropanoic acid	−0.797	16.984	0.00307	0.01222
L-Tyrosyl-L-glutamine	0.694	16.728	0.00317	0.01246
Pyroglutamic acid	0.419	23.178	0.00351	0.01361
sn-Glycerol 3-phosphate	0.544	19.750	0.00409	0.01542
L-Alanine	0.468	21.447	0.00456	0.01700
Thiaminpyrophosphate	0.972	17.802	0.00465	0.01708
L-Tyrosine	1.005	18.152	0.00495	0.01796
Serotonin	−0.856	19.538	0.00630	0.02227
5-Hydroxy-D,L-lysine	−0.541	16.762	0.00655	0.02274
L-Argininosuccinic acid	0.489	20.133	0.00659	0.02274
Uric acid	−0.872	20.411	0.00739	0.02490
L-Prolyl-L-threonine	0.456	17.391	0.00754	0.02491
Caffeic acid	0.846	11.955	0.00757	0.02491
L-Glutamine	0.415	23.021	0.00793	0.02579
gamma-Glutamyl-tryptophan	−1.178	18.306	0.00813	0.02614
S-(2-Carboxyethyl)cysteine	−0.912	19.479	0.00890	0.02799
Cytidine monophosphate	0.449	24.217	0.00890	0.02799
Uridine	−0.650	23.353	0.00964	0.02996
L-Valyl-L-alanine	1.011	18.636	0.01030	0.03168
Spermidine	−0.449	22.564	0.01043	0.03174
Spermine	−1.144	20.609	0.01090	0.03252
Pyruvic acid	0.570	16.746	0.01126	0.03319
(3-Carboxypropyl) trimethylammonium	−0.226	25.234	0.01211	0.03533
Urocanic acid	1.357	20.985	0.01319	0.03809
Glyceraldehyde 3-phosphate	1.960	19.691	0.01448	0.04138
Rutin	1.789	11.194	0.01504	0.04256
Guanosine	−0.551	26.262	0.01548	0.04336
Stearic acid (FA 18:0)	0.412	17.346	0.01606	0.04447
L-Lysyl-L-glutamic acid	−0.465	17.413	0.01619	0.04447
Xanthosine	−0.864	18.294	0.01649	0.04486
Palmitic acid (FA 16:0)	0.457	17.561	0.01711	0.04611
Cytidine 5prime-diphosphoethanolamine	−0.249	23.064	0.01821	0.04817
L-Phenylalanyl-L-threonine	0.550	16.795	0.01842	0.04826
1-Methylnicotinamide	−0.390	19.488	0.01907	0.04951

**Table 3:** List of the 41 KEGG metabolism pathways for which at least three identified metabolites were measured. For each pathway, its name, number of up- and downregulated features, raw *p*-value, and adjusted *p*-value are reported. Based on the adjusted *p*-value, 31 of the listed metabolic pathways were significantly altered in the *Mecp2^−/y^* cortex.

Pathway	Number of Features	Number down	Number up	Direction	*p*-Value	Adjusted *p*-Value	Group
Starch and sucrose metabolism	3	0	3	Up	1.00 × 10^−6^	1.02 × 10^−5^	Carbohydrate metabolism
Glyoxylate and dicarboxylate metabolism	11	0	8	Up	8.00 × 10^−6^	6.97 × 10^−5^	
Galactose metabolism	4	0	4	Up	1.20 × 10^−5^	7.86 × 10^−5^	
Fructose and mannose metabolism	3	0	3	Up	1.40 × 10^−5^	7.91 × 10^−5^	
Glycolysis/Gluconeogenesis	5	0	5	Up	5.40 × 10^−5^	0.000199	
Citrate cycle (TCA cycle)	7	0	7	Up	9.60 × 10^−5^	0.000301	
Pentose phosphate pathway	3	0	3	Up	0.000106	0.000309	
Butanoate metabolism	6	0	6	Up	0.000115	0.000313	
Ascorbate and aldarate metabolism	3	0	3	Up	0.00013	0.000332	
Pentose and glucuronate interconversions	5	0	5	Up	0.000211	0.000508	
Pyruvate metabolism	6	0	6	Up	0.000333	0.000717	
Propanoate metabolism	4	0	3	Up	0.000623	0.001215	
Oxidative phosphorylation	5	0	4	Up	0.000295	0.000671	Energy metabolism
Sulfur metabolism	6	2	4	Up	0.025918	0.034278	
Biosynthesis of unsaturated fatty acids	4	0	3	Up	0.001717	0.002815	Lipid metabolism
Primary bile acid biosynthesis	3	0	2	Up	0.00549	0.008336	
Glycerophospholipid metabolism	4	2	1	Down	0.01947	0.027526	
Pyrimidine metabolism	16	7	3	Down	0.116182	0.136098	Nucleotide metabolism
Purine metabolism	20	7	5	Up	0.257673	0.270886	
Arginine biosynthesis	10	0	8	Up	1.00 × 10^−6^	1.02 × 10^−5^	Amino acid metabolism
Valine, leucine, and isoleucine degradation	3	0	3	Up	9.00 × 10^−6^	6.97 × 10^−5^	
Alanine, aspartate, and glutamate metabolism	12	0	10	Up	1.80 × 10^−5^	8.97 × 10^−5^	
Valine, leucine, and isoleucine biosynthesis	4	0	4	Up	2.50 × 10^−5^	0.0001	
Phenylalanine metabolism	5	0	5	Up	6.20 × 10^−5^	0.00021	
Arginine and proline metabolism	17	3	9	Up	0.000503	0.00103	
Cysteine and methionine metabolism	13	4	9	Up	0.001386	0.00247	
Phenylalanine, tyrosine, and tryptophan biosynthesis	3	0	3	Up	0.00163	0.002784	
Glycine, serine, and threonine metabolism	13	4	7	Up	0.00343	0.005408	
Lysine degradation	7	5	1	Down	0.07318	0.093761	
Histidine metabolism	9	2	3	Up	0.143679	0.163634	
Tyrosine metabolism	7	2	4	Up	0.214087	0.230988	
Tryptophan metabolism	4	1	2	Up	0.377303	0.386735	
D-Glutamine and D-glutamate metabolism	3	0	3	Up	2.00 × 10^−6^	2.05 × 10^−5^	Metabolism of other amino acids
Taurine and hypotaurine metabolism	7	1	5	Up	0.001137	0.002118	
beta-Alanine metabolism	9	4	2	Down	0.113484	0.136098	
Glutathione metabolism	9	4	4	Up	0.184715	0.204683	
Porphyrin and chlorophyll metabolism	3	0	2	Up	2.30 × 10^−5^	0.0001	Metabolism of cofactors and vitamins
Nicotinate and nicotinamide metabolism	8	1	5	Up	0.00984	0.014408	
Thiamine metabolism	8	3	4	Up	0.022741	0.031079	
Vitamin B6 metabolism	3	0	1	Up	0.110406	0.136098	
Pantothenate and CoA biosynthesis	8	3	3	Up	0.505619	0.505619	

## Data Availability

The data presented in this study are available on request from the corresponding author.

## Supplementary Materials

The following are available online at https://www.mdpi.com/article/10.3390/cells10092494/s1, Table S1: List of all annotated metabolites.
