# Supplementary material for: Metabolomic Fingerprint of Mecp2-Deficient Mouse Cortex: Evidence for a Pronounced Multi-Facetted Metabolic Component in Rett Syndrome
Source: Cells. 2021 Sep 21;10(9):2494. doi: 10.3390/cells10092494 (PMC8472238; doi:10.3390/cells10092494)
Supplement: Supplementary file 1 [file cells-10-02494-s001.zip › cells-1359318-supplementary.pdf]

Supplemental Table S1: List of all annotated metabolites.

Note that 32 out of the 283 measurements could not be identified unambiguously, as they were annotated to more than one reference compound.

Metabolite	Log fold change	Average expression	p-value	Adjusted p-value
L-Proline	0.910364847	19.268619	1.02×10^{-6}	0.000143678
L-Glutamic acid	1.405947697	19.8986119	2.04×10^{-6}	0.000182672
N-Acetylglutamic acid	1.14608971	13.49508312	7.33×10^{-7}	0.000143678
Adenosine monophosphate	0.925431723	20.92283765	3.23×10^{-6}	0.000182672
Adenosine diphosphate	0.925431723	20.92283765	3.23×10^{-6}	0.000182672
L-Threonine	1.280944045	18.62842653	9.24×10^{-6}	0.000355332
L-Glutamylglycine;<br>L-Aspartyl-L-alanine;<br>Glycyl-L-glutamic acid;<br>L-Alanyl-L-aspartic acid	-1.884776605	20.42198275	8.92×10^{-6}	0.000355332
L-Leucine	0.869859403	17.67189279	1.18×10^{-5}	0.000355332
D-Glucose 6-phosphate	2.225577112	12.01816324	1.23×10^{-5}	0.000355332
Citric acid	0.872324355	18.51228499	1.38×10^{-5}	0.000355332
Inositol 1-phosphate	0.959271973	13.17443792	1.45×10^{-5}	0.000355332
(S)-Methyl-3-hydroxybutanoate	0.934581762	16.36377358	1.51×10^{-5}	0.000355332
Putrescine	1.178846798	14.87831494	2.05×10^{-5}	0.000441438
D-Fructose 1,6-bisphosphate	2.035249928	12.80678239	2.18×10^{-5}	0.000441438
D-Fructose	1.167056757	15.98535056	2.89×10^{-5}	0.000546067
L-Valine	0.787206983	18.17950115	3.55×10^{-5}	0.00060476
L-Isoleucine	0.79431364	16.5102761	3.63×10^{-5}	0.00060476
L-Homocysteine	-2.331632788	19.23053362	3.89×10^{-5}	0.000611821
Inosine 5prime-monophosphate	1.157600877	15.51293914	4.43×10^{-5}	0.000659335
D-Glucose	1.418283415	14.89091169	5.85×10^{-5}	0.000800649
Urea	1.598661908	18.09257677	6.27×10^{-5}	0.000800649
L-Glutamyl-L-glutamine	-1.884904713	27.11648733	6.07×10^{-5}	0.000800649
5prime-Deoxy-5prime-(methylthio)adenosine	0.732141245	12.14648557	6.81×10^{-5}	0.000800649
(5Z,8Z,11Z,14Z)-Icosatetra-5,8,11,14-enoic acid	1.031702338	12.83350372	6.63×10^{-5}	0.000800649
L-Rhamnose	0.805086123	15.91103464	7.07×10^{-5}	0.000800649
S-Adenosyl methionine	-0.993747197	23.74037586	9.39×10^{-5}	0.001021811
L-Threonic acid	0.684850562	16.7441406	0.000100839	0.001056943
Xanthine	-0.590421827	24.31018454	0.000111038	0.001122275
Glycerol	0.74255619	19.06670092	0.000130275	0.001228923
Uracil	-0.903421442	21.18827062	0.000127396	0.001228923
L-Serine	0.579756565	20.90098081	0.000144906	0.001281509
L-Phenylalanine	1.142606217	16.6882559	0.000143034	0.001281509
Riboflavin	-1.160626938	20.07087316	0.000183039	0.001569698

Erythritol	0.69635314	13.02246792	0.000213423	0.001776433
Orthophosphate	0.611876768	20.97105837	0.000287398	0.002323819
L-Dehydroascorbic acid	0.691235622	21.84685323	0.000323978	0.002522096
alpha-Ketoglutaric acid	1.782703728	12.7661811	0.000331765	0.002522096
Succinic acid	0.742693312	17.42949611	0.000338656	0.002522096
L-Methionine	0.908894628	16.36105358	0.000407954	0.002960279
1-Methyl-4-Imidazoleacetic acid	-0.837075378	21.80343026	0.000443631	0.003119355
Dopamine	-1.284697775	21.78807503	0.000462943	0.003119355
D-Mannose;<br>D-Tagatose;<br>D-Galactose;<br>myo-Inositol;<br>allo-Inositol;<br>alpha-D-Talose;<br>D-chiro-Inositol	-1.039213853	25.74456344	0.000456768	0.003119355
Choline	-0.431043703	26.90171757	0.000505721	0.00332835
L-Malic acid	0.694670848	17.42147725	0.00060495	0.003890931
alpha-D-Glucose 1-phosphate	0.608475547	16.33371138	0.000649924	0.004087301
Acetylcholine	-0.927272907	23.07065289	0.000673763	0.004145105
Guanidineacetic acid	0.820712568	20.09857657	0.000800676	0.004799821
Cholesterol	1.26134407	19.0671946	0.000814104	0.004799821
gamma-Glutamyl-tyrosine	-1.785159912	19.52425254	0.000842754	0.004867336
L-Phenylalanyl-L-glutamic acid	0.897985638	18.32305035	0.000890862	0.004972086
L-Tryptophan	1.611387398	14.88326519	0.00089603	0.004972086
L-Glutamyl-L-Serine;<br>L-Aspartyl-L-threonine;<br>L-Seryl-L-glutamic acid;<br>L-Threonyl-L-aspartic acid	-1.150295438	19.73175287	0.000937591	0.005102657
Sucrose	6.158042428	13.3923006	0.001012537	0.005406564
3-Methoxytyramine	-1.329118853	19.23131033	0.001059866	0.005554481
Hydroxymethylphosphonic acid	-0.787341612	23.67877477	0.001146676	0.005900169
Cysteinylglycine	-4.027560695	21.77167408	0.001183926	0.005983054
L-Cystine	0.873750312	11.92833169	0.00122662	0.00600919
L-Lysine	-0.351196902	24.20427702	0.001231565	0.00600919
Xylitol	0.808853428	14.89013391	0.001398548	0.006708289
L-Valylglycine	0.679369707	18.35627748	0.001506708	0.00706882
myo-Inositol 2-phosphate	0.595473178	12.66808989	0.001523668	0.00706882
Quinic acid	0.855868818	11.41828108	0.001572353	0.007144912
Pantothenic acid	0.684616238	13.36373728	0.001590564	0.007144912
gamma-Glutamyl-leucine	-0.625316557	20.67488703	0.001683805	0.007445575
L-Homoserine	0.68975963	13.17477756	0.002011208	0.008756489
L-Tryptophyl-L-glutamic acid	1.066921912	17.06268455	0.002429858	0.010418938
O-Acetyl-L-homoserine	-0.757940773	25.05720935	0.00256228	0.010822766
2-Hydroxypyridine	0.676439085	18.51302151	0.002669588	0.011110195
L-Tyrosylglycine	0.668551912	17.22759761	0.002757835	0.01131112
Cytidine	-0.498261918	24.93982939	0.002828586	0.011435569

3-Ureidopropanoic acid	-0.797498453	16.98390769	0.003066315	0.012222074
L-Tyrosyl-L-glutamine	0.694020544	16.72809727	0.003170807	0.012463033
Pyroglutamic acid	0.419081783	23.17766854	0.00350977	0.013606368
Homoserine lactone;<br>1-Aminocyclopropanecarboxylic acid	0.74211736	23.15446502	0.00381263	0.014580734
sn-Glycerol 3-phosphate	0.543551875	19.7500315	0.004085449	0.01541576
L-Alanine	0.468433877	21.44703378	0.004564538	0.016996897
Thiamine pyrophosphate	0.971660353	17.80216496	0.004646168	0.017076178
L-Tyrosine	1.004840045	18.15237569	0.004949104	0.017956364
D-Gluconic acid;<br>D-Galactonic acid	-0.57716358	21.82861582	0.005566274	0.019939945
Serotonin	-0.856307127	19.53843285	0.006295366	0.022269858
5-Hydroxy-D,L-lysine	-0.540744435	16.76212247	0.006548181	0.022737002
L-Argininosuccinic acid	0.488731305	20.13284785	0.006588107	0.022737002
L-Carnosine;<br>L-Histidylalanine;<br>L-Alanyl-L-histidine	-0.510078428	23.84237731	0.006742973	0.022991102
Uric acid	-0.871941502	20.41143074	0.007390704	0.024899634
L-Prolyl-L-threonine	0.456035648	17.39143136	0.007544332	0.024909985
Caffeic acid	0.846467135	11.9550661	0.007569819	0.024909985
L-Glutamine	0.414533575	23.02109404	0.007928206	0.025789453
gamma-Glutamyl-tryptophan	-1.177923893	18.30634829	0.008127535	0.026137415
S-(2-Carboxyethyl)cysteine	-0.91157588	19.47881657	0.008900643	0.027987579
Cytidine monophosphate	0.4489582	24.21657427	0.008898191	0.027987579
Uridine	-0.649567915	23.35256602	0.009635198	0.029964406
L-Valyl-L-alanine	1.01063017	18.6355412	0.01029879	0.031679973
Spermidine	-0.449078377	22.56408946	0.010430484	0.031740074
D-Fructose 1-phosphate;<br>D-Fructose-6-phosphate;<br>myo-Inositol 2-phosphate;<br>alpha-D-Mannose 1-phosphate;<br>alpha-D-Glucose 1-phosphate;<br>alpha-D-Glucose 6-phosphate;<br>alpha-D-Galactose 1-phosphate	0.558820403	22.05377729	0.01091753	0.032522746
Spermine	-1.143593858	20.60944719	0.010896321	0.032522746
Pyruvic acid	0.570419873	16.74637866	0.011257659	0.033186641
(3-Carboxypropyl)trimethylammonium	-0.226471368	25.23419337	0.012109628	0.035330153
Urocanic acid	1.356715892	20.98518796	0.013189	0.038086602
Glyceraldehyde 3-phosphate	1.960244427	19.69145219	0.014475453	0.041379325
Rutin	1.788608378	11.19429253	0.015037426	0.042555916
Guanosine	-0.55096833	26.26155349	0.015475131	0.04336101
Stearic acid (FA 18:0)	0.41242329	17.34639851	0.016057562	0.044469661
L-Lysyl-L-glutamic acid	-0.464667625	17.41251084	0.016185071	0.044469661
Xanthosine	-0.864172112	18.29395621	0.01648527	0.044858955
Palmitic acid (FA 16:0)	0.457120538	17.56149856	0.01710802	0.046110188

N-Acetyl-D-glucosamine;<br>N-Acetyl-D-mannosamine;<br>N-Acetyl-D-galactosamine	-0.412035985	18.02620754	0.018034925	0.048149846
Cytidine 5prime-diphosphoethanolamine	-0.248695498	23.06352172	0.018211465	0.048166773
L-Phenylalanyl-L-threonine	0.549963989	16.79472457	0.018417025	0.048259427
1-Methylnicotinamide	-0.389940475	19.48795031	0.019070126	0.049512345
L-Tyrosyl-L-Threonine	0.78708422	18.58742469	0.019937919	0.051294828
alpha-L-Fucose 1-phosphate	0.43322505	18.82915783	0.021782108	0.055194714
L-Tyrosyl-L-glutamic acid	0.325103968	17.19357652	0.021843844	0.055194714
Nicotinic acid	0.758012388	20.2361408	0.022382698	0.056055784
N-epsilon-Acetyl-L-lysine	-0.432392877	20.49597802	0.022892564	0.056829787
Inosine	-0.317629925	28.07012108	0.024738424	0.060878034
L-Isoleucyl-L-glutamine	0.408583998	18.67223619	0.026528608	0.064720656
Cytosine	-0.379539169	18.84250837	0.027579068	0.066334106
S-Adenosyl-L-homocysteine	0.791297963	21.7158033	0.027720531	0.066334106
O-Phospho-L-serine	-0.377281948	21.56297239	0.02789314	0.066334106
L-Ornithine	0.716137403	19.14741915	0.028297154	0.066639605
(2S)-2-amino-3-(1-Methyl-1H-imidazol-5-yl)propanoic acid	-0.435924287	20.76259687	0.028492552	0.066639605
Pyridoxal-5prime-monophosphate	0.575858427	19.11444236	0.02931851	0.068009331
Suberic acid	0.739782503	16.71331223	0.029887359	0.068765225
Glycyl-L-phenylalanine	-0.374836698	18.53844512	0.030537511	0.069694482
Cystathionine	0.619857418	22.28752207	0.033281649	0.075349652
Adenine	-0.224482767	25.29713844	0.037077221	0.083276616
N-epsilon,N-epsilon,N-epsilon-Trimethyllysine	-0.210268312	24.21061457	0.049981248	0.111375536
L-Prolylglycine	0.36533213	18.89061966	0.053364919	0.117825334
Cytidine diphosphate	-0.958696578	18.33000485	0.053708368	0.117825334
5-(2-Hydroxyethyl)-4-methylthiazole	-0.309122134	16.61242141	0.054746399	0.1191787
Betaine	-0.426471245	24.25424968	0.055950079	0.120869255
3-Hydroxy-3-methylglutaric acid	-0.464477942	18.44493994	0.058394456	0.125194174
Azelaic acid	0.757162352	18.9047093	0.059179291	0.125922852
Sorbitol	3.448546223	13.67641652	0.060048797	0.126819475
Benzoic acid	0.591421583	15.037138	0.060626132	0.126986652
4-Guanidinobutyric acid	0.443544658	20.49088725	0.061025387	0.126986652
D-Maltose;<br>D-Trehalose	2.790938455	19.70644242	0.064000538	0.13220549
L-Lysyl-L-serine;<br>L-Seryl-L-lysine	0.370505672	16.91285605	0.066391527	0.13615074
D-Ribose 5-phosphate	0.414422035	13.65919217	0.0674557	0.136504507
L-Threonyl-L-valine	0.775123767	17.77678373	0.068171048	0.136504507
4-Aminobutanoic acid	0.24786664	22.31316696	0.068439641	0.136504507
(S)-Lactate	0.228093765	23.79639063	0.068493428	0.136504507
Uridine 5-diphosphoglucose;<br>Uridine 5prime-diphosphogalactose	-0.239379808	24.9095793	0.069478064	0.137498546
L-Glutamyl-L-threonine	0.302300685	18.44118959	0.070974493	0.139484593

2-aminoadipate	0.800863145	15.31156504	0.079029569	0.154243917
L-Seryl-L-leucine	0.507236279	17.31328703	0.080826953	0.156671423
(S)-Piperidine-2-carboxylic acid	-0.454693462	21.40476645	0.081496688	0.156894984
L-Kynurenine	0.621790223	18.69611315	0.08235143	0.156991328
N-Acetyl-L-methionine	-0.720368362	18.78402303	0.08307763	0.156991328
Deoxyguanosine	-0.357037316	16.85451565	0.083210951	0.156991328
L-Methionyl-L-glutamic acid	0.274872002	16.74667274	0.0858921	0.160976585
L-Alanyl-L-leucine	0.490342713	17.15252343	0.08684978	0.161700577
D-Ribose 1-phosphate;<br>D-Ribulose 5-phosphate;<br>alpha-D-Xylose 1-phosphate	0.355405053	21.99715189	0.093031023	0.17207699
Fumaric acid	0.370239998	17.56798248	0.095677251	0.175822481
Uridine diphosphate	-0.205964832	22.19805715	0.097170764	0.177415008
Panose;<br>Maltotriose;<br>D-Melezitose	-0.39428218	18.18038869	0.098059572	0.177890122
Taurine	0.251256092	21.62100626	0.101238925	0.182487998
Tetronic acid	-0.281660618	20.82036732	0.109560352	0.196237846
Cytidine 3prime-phosphate	-0.2037424	20.86691334	0.110263807	0.196255706
L-Cycloserine	-0.21864199	18.90000514	0.116639615	0.206306319
L-Leucyl-L-glutamic acid	0.222366043	19.08364456	0.123818511	0.217643718
Saccharopine	-0.426239362	21.28423647	0.132098064	0.229672035
L-Cysteine	-0.38604679	16.9879281	0.1322846	0.229672035
Allantoin	-0.25628377	18.58204082	0.140543664	0.242523517
L-Prolyl-L-serine	0.486899265	17.88428183	0.143067518	0.24538247
Thiamine	-0.234791328	18.44618401	0.144611556	0.245483696
2-Methylcitric acid	-0.305009885	16.9187475	0.145075223	0.245483696
Mannitol;<br>Sorbitol;<br>Galactitol	2.002549017	19.77159292	0.145728837	0.245483696
6-Phosphogluconic acid	0.347349115	19.85087911	0.150129365	0.251400061
O-Acetyl-L-carnitine	-0.141578918	28.24508256	0.155731921	0.259247846
L-Seryl-L-aspartic acid	0.271594752	16.73033434	0.158157217	0.261745569
Adenosine 5prime-diphosphoglucose	-0.277176755	17.20260742	0.166179913	0.273423927
L-Isoleucyl-L-arginine	-0.446784308	18.05843498	0.169988453	0.278073596
L-Lysylglycine	0.257015302	17.3295692	0.175414066	0.285299889
Glycyl-L-valine	-0.25532188	17.05209613	0.178605212	0.288830143
Thymidine	-0.272594763	17.30096348	0.180317258	0.289941955
1-Methylhistamine;<br>3-Methylhistamine	-0.341831945	18.61035738	0.181401284	0.290037081
L-Alanyl-L-threonine;<br>L-Threonyl-L-alanine	-0.216994242	19.7337492	0.184213091	0.292878117
Glycyl-L-tyrosine	-0.264798088	19.1262099	0.185869669	0.293860984
Glycerol-3-phosphate;<br>beta-Glycerophosphate	-0.121939528	24.44080082	0.199042619	0.312939229

5-Aminolevulinic acid;<br>cis-4-Hydroxy-D-proline;<br>trans-3-Hydroxy-L-proline;<br>trans-4-Hydroxy-L-proline	-0.273002058	20.58611923	0.207912842	0.325079194
Nonanoic acid	0.321176343	11.63150154	0.213054657	0.331288285
Oxalate	0.221267312	16.88292015	0.237289785	0.366956334
2-Aminoethyl-dihydrogen phosphate	0.388049553	25.44811468	0.243597951	0.374664239
Thymidine 5prime-phosphate	0.132874713	17.61013028	0.250409932	0.383059517
N-Acetylneuraminic acid	-0.127697747	24.03802202	0.252638078	0.384390193
Deoxyuridine	-0.19656435	17.19592557	0.256509658	0.38819376
L-Aspartylglycine	0.254090233	18.37873498	0.261684722	0.393919023
N,N-Dimethylglycine;<br>3-Aminoisobutyric acid;<br>(R)-2-Aminobutanoic acid	0.240180163	27.43599785	0.267219642	0.398016625
N-Acetyl-L-cysteine	-0.554378058	17.32169002	0.267209223	0.398016625
Stachyose;<br>Maltotetraose	-0.306227958	17.26257436	0.27412469	0.406163807
Adenosine 3prime,5prime-cyclic phosphate	-0.5090874	19.6299343	0.279465236	0.410763816
Creatine	-0.107361217	32.08303922	0.280132214	0.410763816
Glycine	0.157526417	21.21737034	0.286478802	0.417904644
Hypoxanthine	0.371336537	25.77080639	0.2935553	0.426031538
L-Valyl-L-asparagine	0.246238065	16.7975536	0.298468244	0.430951597
L-Carnitine	-0.114893328	28.46279473	0.301372709	0.432936429
L-Leucylglycine	0.559558567	19.25290275	0.314710374	0.449813312
Lignoceric acid (FA 24:0)	0.287638793	10.76693625	0.31650514	0.450105299
2-[[Amino(imino)methyl]amino]ethane-1-sulfonic acid	0.164176855	18.62501903	0.327624525	0.46167277
L-Seryl-L-isoleucine	0.209295507	18.27821313	0.328891416	0.46167277
[(3aS,4S,5S,6E,10E,11aR)-6-formyl-5-methoxy-10-methyl-3-methylidene-2-oxo-3a,4,5,8,9,11a-hexahydrocyclodeca[b]furan-4-yl] 2-methylpropanoate	0.197472753	17.40360234	0.330114489	0.46167277
L-Phenylalanyl-L-glutamine	0.220418978	16.87019526	0.331164566	0.46167277
L-Aspartic acid;<br>Iminodiacetic acid	-0.119727602	27.93348998	0.33693424	0.467413676
L-Alanyl-L-isoleucine	0.182288852	17.15324193	0.34990719	0.483042609
beta-Alanine	0.217722758	14.10682074	0.367332595	0.504636527
Phosphocreatine	0.140266642	23.77139433	0.376821285	0.511049118
Hexacosanoic acid	0.175616915	12.9437497	0.377347303	0.511049118
Pyridoxal	0.347706888	18.40045789	0.378158032	0.511049118
Nobiletin	0.14347851	19.22033348	0.379223727	0.511049118
2prime-Deoxycytidine-5prime-monophosphate	0.145182583	18.43209072	0.392123443	0.525928599
Ascorbic acid	-0.099171523	27.94229016	0.396346491	0.529085174

L-Glutamyl-L-alanine;<br>L-Alanyl-L-glutamic acid	0.287383178	19.95252448	0.406885184	0.540603319
Glycyl-L-isoleucine	0.223383035	16.6444878	0.416671462	0.551018803
L-Alanyl-L-valine	-0.31406529	19.26137989	0.422242005	0.555788313
Maleamic acid	-0.06879747	22.60856983	0.427301208	0.559525313
p-Hydroxyphenyllactic acid	0.27287364	20.56651076	0.42903531	0.559525313
Sedoheptulose-7-phosphate;<br>(D-Glycero-alpha-D-Manno-<br>Heptopyranosyl)-Dihydrogenphosphate	0.111447815	19.1681878	0.444219317	0.574036834
L-Alanyl-L-glutamine	-0.207188794	17.60715722	0.44391952	0.574036834
Allantoic acid	0.24635466	13.07021586	0.447274272	0.575357359
Sorbitol-6-phosphate	0.295087782	17.91562592	0.4600889	0.589163614
L-Isoleucyl-L-serine	0.200925562	18.3470923	0.470028255	0.599180163
myo-Inositol	0.182975533	20.91555376	0.486289259	0.617129419
Histamine	0.146385397	16.34509258	0.49194699	0.621522313
Anserine	-0.181563823	23.59572665	0.495673649	0.6234473
5,6-Dihydrothymine	-0.224879488	17.26050037	0.499171966	0.62506932
Guanosine diphosphate	0.21777941	22.69904781	0.510691852	0.636677507
L-Alanyl-L-alanine;<br>Gly-gamma-aminobutyric acid	-0.079968801	20.24646151	0.517940688	0.642882521
L-Valyl-L-glutamine	0.087451776	18.52988981	0.525407519	0.648619195
Benzophenone	0.097047613	17.61253914	0.528296157	0.648619195
L-Alanyl-L-tyrosine;<br>L-Phenylalanyl-L-serine	0.224084115	17.78636549	0.529438283	0.648619195
N-Acetyl-D-phenylalanine	0.28269008	16.8647516	0.538934165	0.656956186
L-Isoleucyl-L-alanine	-0.266186107	19.33500414	0.5446054	0.656956186
Guanine	-0.08556109	18.02553266	0.546826021	0.656956186
L-Seryl-L-valine	0.192702903	19.14474587	0.546870536	0.656956186
L-beta-Imidazolelactic acid	0.209643963	19.10859778	0.547850388	0.656956186
Geraniol;<br>Fenchyl alcohol	0.11687557	21.19447782	0.562977834	0.672247793
Creatinine	0.047433208	24.43087274	0.576468403	0.685464529
N-Acetyltyrosine	-0.15975452	16.22813998	0.606162019	0.7177567
Ergothioneine	0.072955258	22.09382041	0.621211881	0.732279232
L-Isoleucyl-L-aspartic acid	-0.102294241	17.40200217	0.628338401	0.732279232
L-Valyl-L-histidine	0.132541948	16.49600753	0.628269178	0.732279232
Pyridoxamine-5prime-phosphate	0.110000037	20.01350748	0.629110288	0.732279232
Tangeretin	0.080246118	19.12312298	0.631364426	0.732279232
Nicotinamide	0.05197329	28.86532817	0.637856422	0.736789255
L-Histidine	-0.063566577	24.42788823	0.654229165	0.752629487
L-Valyl-L-lysine	-0.036907908	21.86901084	0.673616257	0.771795145
L-Leucyl-L-alanine	0.111360405	16.93668639	0.678295359	0.774022527
L-Isoleucyl-L-threonine	0.056888057	17.42436146	0.704701429	0.792312209
L-Isoleucyl-L-isoleucine	-0.102214095	16.71705052	0.707152581	0.792312209
L-Glutamyl-L-valine;<br>L-Leucyl-L-aspartic acid	-0.060355131	17.8925777	0.708309945	0.792312209

beta-Nicotinamide-adenine dinucleotide	0.100652088	17.10216725	0.709180583	0.792312209
Deoxycytidine	0.048201747	19.194098	0.711084108	0.792312209
L-Aspartyl-L-glutamine;<br>L-Asparaginyl-L-glutamic acid	-0.072020438	19.28502195	0.711121205	0.792312209
5-Hydroxyindole-3-acetic acid	0.070323523	19.53104177	0.72124195	0.800437145
L-Isoleucyl-L-lysine	0.068316414	16.62637454	0.727835673	0.803147187
L-Valyl-L-serine	-0.104640942	18.41354809	0.729359813	0.803147187
L-Prolyl-L-glutamic acid	0.042081428	19.8964025	0.752536558	0.825456767
L-Leucyl-L-threonine	0.10189654	17.18343701	0.758964084	0.829292802
L-Arginine	-0.045078635	26.08503146	0.779394761	0.848341221
trans-4-Hydroxy-L-proline	0.094319125	13.91254925	0.788708359	0.855189523
D-Saccharic acid	0.064947098	23.28066932	0.806169823	0.870786489
D-Panthenol	-0.233323028	19.27129437	0.826433082	0.889279704
L-Glutamyl-L-aspartic acid	0.032933153	18.30391916	0.836559484	0.896766416
4-Pregnene-11-beta-17-alpha-diol-3-20-dione;<br>(9xi,11beta,14xi)-11,21-Dihydroxypregn-4-ene-3,20-dione	-0.041279007	16.93100044	0.864817922	0.923560271
L-Citrulline	0.024327373	21.06589883	0.869914755	0.925510811
Phosphoglycolic acid	0.028960261	16.66548934	0.879497897	0.931289502
Hypotaurine	-0.031148133	20.28218628	0.881927868	0.931289502
2-Deoxyadenosine 5-phosphate	0.02375839	18.07875758	0.889021997	0.935290799
L-Isoleucyl-L-glutamic acid	-0.014868483	17.76992983	0.901587172	0.944996925
Nerolidol	0.017408927	18.73242769	0.907819996	0.948018667
L-Valyl-L-aspartic acid	-0.016311975	18.73588535	0.930616698	0.968251933
D-Galactose;<br>myo-Inositol;<br>allo-Inositol;<br>muco-Inositol;<br>alpha-D-Talose;<br>D-chiro-Inositol	-0.015170368	24.65683218	0.935056563	0.969307719
5-Aminoimidazole-4-carboxamide-1-beta-D-ribofuranosyl	0.017776888	18.38152973	0.943904694	0.972917594
N-Methylnicotinic acid	-0.016113135	22.60709829	0.94544463	0.972917594
L-Threonyl-L-isoleucine	0.019577629	16.82985874	0.948852494	0.972917594
D-2-Aminoadipic acid;<br>N-Methyl-L-glutamic acid	0.018391918	24.25602673	0.960639228	0.981447298
L-Asparagine	-7.25×10^{-16}	20.26068609	1	1
N-Acetyl-L-aspartic acid	0.001246033	29.29685449	0.988965369	1
2-Aminophenol	-6.01×10^{-18}	17.31210201	1	1
L-Valyl-L-glutamic acid	-0.001462613	19.15888345	0.99457281	1
L-Phenylalanyl-L-valine	-4.85×10^{-15}	16.96386906	1	1
D-Trehalose;<br>D-Gentiobiose	-2.67×10^{-15}	19.29362673	1	1
